# EMBRACING THE DIVERSITY OF GRANDFAMILIES: UNDERSTANDING THE INFLUENCE OF CULTURE AND RACISM

**DOI:** 10.1093/geroni/igac059.1030

**Published:** 2022-12-20

**Authors:** Nancy Mendoza, Loriena Yancura

## Abstract

In line with this year’s conference theme, we examine diversity in different forms, such as racial, cultural, and familial diversity. We discuss the importance of diversity’s influence on the experiences of grandfamilies and the impact it has on their members. Findings from a study with Latinx grandparent caregivers suggest that those raising grandchildren often do not view themselves as “raising,” but instead “helping.” Such findings highlight the importance of understanding cultural norms to appropriately tailor services and resources. In an exploration of the experiences of Korean custodial grandparents, findings demonstrated the influence of patrilineality and stigma surrounding divorce for Korean grandparent-headed families, indicating the importance of considering a grandparents’ position in the family when providing services. In a study with custodial grandparents during the COVID-19 pandemic, researchers examined the role of racial discrimination on grandparents' depressive symptoms and access to health services. Results indicated a higher level of perceived racial discrimination was associated with more depressive symptoms. These results imply the need to address racial/ethnic disparities experienced by these caregivers. Similarly, in a study of emerging adults raised by grandparents, race moderated the influence of attachment on symptoms of racial discrimination. Findings support the use of interventions addressing attachment and ethnic identity to decrease symptoms of racial trauma in grandfamilies. Together these four studies reiterate the diversity of grandfamilies, enrich our understanding of these families, and encourage us to reimagine how to best serve them.

